# Microbial enhanced heavy crude oil recovery through biodegradation using bacterial isolates from an Omani oil field

**DOI:** 10.1186/s12934-015-0330-5

**Published:** 2015-09-16

**Authors:** Abdullah Al-Sayegh, Yahya Al-Wahaibi, Saif Al-Bahry, Abdulkadir Elshafie, Ali Al-Bemani, Sanket Joshi

**Affiliations:** Department of Petroleum and Chemical Engineering, College of Engineering, Sultan Qaboos University, Muscat, Oman; Department of Biology, College of Science, Sultan Qaboos University, Muscat, Oman; Central Analytical and Applied Research Unit, College of Science, Sultan Qaboos University, Muscat, Oman

**Keywords:** Biodegradation, Biotransformation, Microbial enhanced oil recovery, Heavy crude oil, *B. subtilis*, *B. licheniformis*

## Abstract

**Background:**

Biodegradation is a cheap and environmentally friendly process that could breakdown and utilizes heavy crude oil (HCO) resources. Numerous bacteria are able to grow using hydrocarbons as a carbon source; however, bacteria that are able to grow using HCO hydrocarbons are limited. In this study, HCO degrading bacteria were isolated from an Omani heavy crude oil field. They were then identified and assessed for their biodegradation and biotransformation abilities under aerobic and anaerobic conditions.

**Results:**

Bacteria were grown in five different minimum salts media. The isolates were identified by MALDI biotyper and 16S rRNA sequencing. The nucleotide sequences were submitted to GenBank (NCBI) database. The bacteria were identified as *Bacillus subtilis* and *B. licheniformis*. To assess microbial growth and biodegradation of HCO by well-assay on agar plates, samples were collected at different intervals. The HCO biodegradation and biotransformation were determined using GC-FID, which showed direct correlation of microbial growth with an increased biotransformation of light hydrocarbons (C_12_ and C_14_). Among the isolates, *B. licheniformis* AS5 was the most efficient isolate in biodegradation and biotransformation of the HCO. Therefore, isolate AS5 was used for heavy crude oil recovery experiments, in core flooding experiments using Berea core plugs, where an additional 16 % of oil initially in place was recovered.

**Conclusions:**

This is the first report from Oman for bacteria isolated from an oil field that were able to degrade and transform HCO to lighter components, illustrating the potential use in HCO recovery. The data suggested that biodegradation and biotransformation processes may lead to additional oil recovery from heavy oil fields, if bacteria are grown in suitable medium under optimum growth conditions.

## Background

Depletion of light oil resources, increasing global energy demand, abundance of HCO resources and costly enhanced oil recovery (EOR) methods necessitate the development of less costly recovery technology to utilize the heavy oil resources. Microbial enhanced oil recovery (MEOR) methods require less energy input to produce bioactive compounds, which are not affected by global crude oil prices. In comparison to other conventional EOR technologies, MEOR is an economically and an environmentally friendly technology [1, 2]. Economically, the oil recovery costs of deep-water fields with gas and water injection is 3–4 USD/barrel. The cold production cost in conventional heavy oil sands is 6 USD/barrel and for hot/steam 17 USD/barrel. The production cost of hydrocarbon by microorganisms is 1.4–2.4 USD/barrel [3]. MEOR through biodegradation or biotransformation of crude oil is the process of converting heavy ends of petroleum crude oil to lighter compounds. Due to their versatility, bacteria play a major role during biotransformation of hydrocarbons; however, there is no single species that will completely degrade any complex class of hydrocarbons [4]. There are more than 175 reported genera of bacteria which utilize hydrocarbons only or when mixed with other nutrients [5]. Bacteria produce different compounds like biosurfactants, enzymes, emulsifiers, etc., which help in the uptake and biodegradation of non-aqueous phase liquids (NAPL) like crude oils [6, 7]. Direct cell contact and biosurfactant-mediated interfacial tension reduction with emulsified hydrocarbons are the general mechanisms of hydrocarbon biodegradation [8]. The majority of MEOR research is focused on crude oil viscosity reduction by means of microbial metabolites; however, not much attention has been given to the direct microbial degradation of heavy oil [9]. During biodegradation and biotransformation processes, microorganisms could produce more than one bioproduct to facilitate crude oil viscosity reduction [10], wherein employing more than one bacterial strain could enhance biodegradation even further [11]. Even toxic asphalt and aromatics compounds are subject to biodegradation [12–14]. Generally, microbial growth on hydrocarbons under anaerobic conditions is considerably slower in comparison with aerobic conditions [15]. Nevertheless, that does not eliminate the possibility of having comparable results in aerobic and anaerobic conditions [10]. The objectives of this study were to isolate bacteria present locally at an Omani oil field that are able to biodegrade and biotransform HCO to enhance its recovery.

## Results and discussion

### Isolation and screening of heavy crude oil biodegrading bacteria

Microbial cultures on agar plates containing HCO (968 cP viscosity; 0.961 g/cc density at 40 °C) showed that colonies ranging from few to many able to biodegrade heavy oil at 40 °C after 10 days of incubation. Five different types of reported minimal media, M1–M5 (Table 1), were used for initial screening of bacterial isolates capable to grow on only heavy oil, where M2, M4 and M5 media supported better growth and strong biodegradation. Whereas both media M1 and M3 neither supported growth, nor HCO biodegradation. The rest of the experiments were performed on M2, M4 and M5 only. In total, 17 bacterial strains were isolated: 12 from M2 (AS1–AS10, AS16 and AS17), 3 from M4 (AS11–AS13) and 2 from M5 (AS14 and AS15). Only spore forming bacteria were isolated, and screened in all of the experiments.

**Table 1:** The minimal media compositions

Compounds (g/L)	M1	M2	M3	M4	M5
	Ref. [11]	Ref. [21]	Ref. [8]	Ref. [19]	Ref. [20]
CaCl_2_	0.0275	–	–	0.00077	–
CaCl_2_·2H_2_O	–	–	0.14	–	0.02
FeCl_3_	–	–	–	–	0.05
FeCl_3_·6H_2_O	0.025	–	–	–	–
FeSO_4_·7H_2_O	–	–	–	0.0011	–
KCl	–	–	0.34	–	–
KH_2_PO_4_	0.085	1.0 g/L	–	4.08	1.0
K_2_HPO_4_	0.21	1.0 g/L	0.14	–	1.0
KNO_3_	–	0.5 g/L	–	–	–
MgCl_2_·6H_2_O	–	–	1.0	–	–
MgSO_4_·7H_2_O	0.0225	0.5 g/L	–	0.2	0.2
MnSO_4_·4H_2_O	–	–	–	0.00067	–
NaCl	–	–	15.0	–	–
Na_2_HPO_4_·2H_2_O	0.33	–	–	–	–
Na_2_HPO_4_	–	–	–	7.12	–
Na-EDTA	–	–	–	0.00148	–
NH_4_Cl	0.005	–	0.25	–	–
NH_4_NO_3_	–	–	–	4.0	1.0
Yeast extract	–	0.5	2.0	–	–
Trace elements	–	10 mL	–	–	–
Vitamin solution	–	1.0 mL	–	–	–

**Fig. 1:**
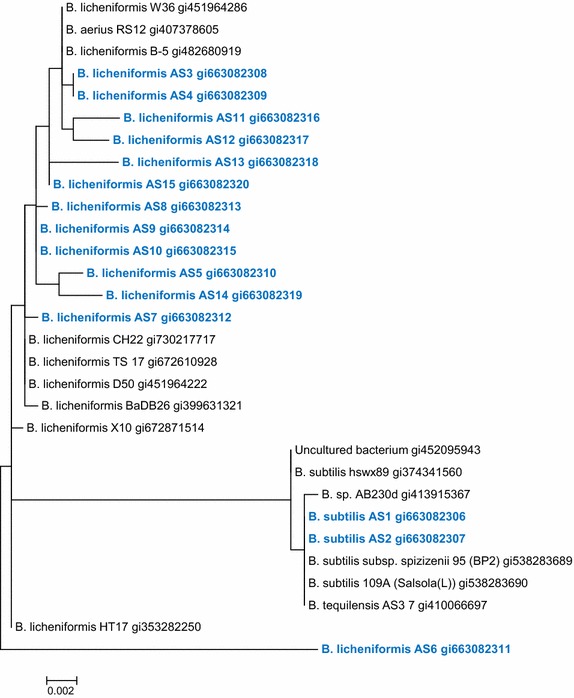
Phylogenetic analysis using maximum likelihood method for bacterial isolates AS1–AS15 [GenBank: KJ729814–KJ729828]

The isolated strains were identified by MALDI biotyping (Bruker MALDI Biotyper CA System). AS1, AS2 and AS6 were identified as *B. subtilis*, AS16 as *B. cereus*, AS17 as *Brevibacillus borstelensis* and the remaining bacteria as *B. licheniformis*. The biotyper has four scoring/confidence categories: highly probable species, probable species (secure genus), probable genus and unreliable identifications. The results were of the probable species (secure genus) identification category. AS16 and AS17 isolates, due to their weak biodegradability performance, were excluded from this study. The remaining 15 bacterial strains were identified using 16S rRNA sequencing. The sequences of AS1 to AS15 were deposited at NCBI GenBank [GenBank: KJ729814 to KJ729828]. AS1 and AS2 were identified as *B. subtilis* and AS3 to AS15 as *B. licheniformis*. Both methods resulted in finding similar species with the exception of AS6 where the biotyper identified it as *B. subtilis* while the molecular sequencing identified as *B. licheniformis* and therefore was considered as *B. licheniformis*. Maximum likelihood method based on the Hasegawa–Kishino–Yano model for the isolates evolutionary history was used to construct phylogenetic tree of the isolates (Fig. 1) [16].

### M2 medium stands out at growth and biodegradation/biotransformation studies

**Fig. 2:**
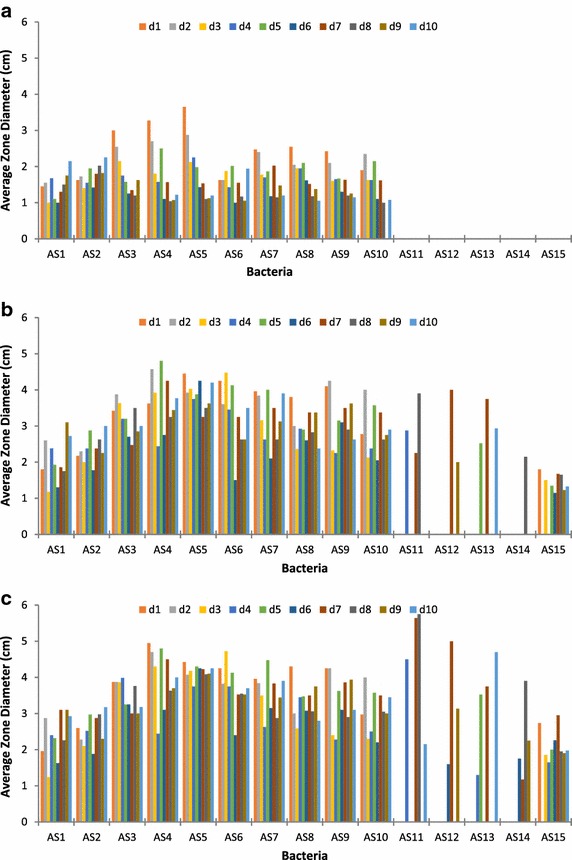
The clearance zones during well-assays for 10 days, for all the isolates after **a** 20, **b** 50 and **c** 70 h. The bacterial strains AS1–AS10 were grown in medium M2, strains AS11–AS13 in medium M4 and strains AS14–AS15 in medium M5

**Fig. 3:**
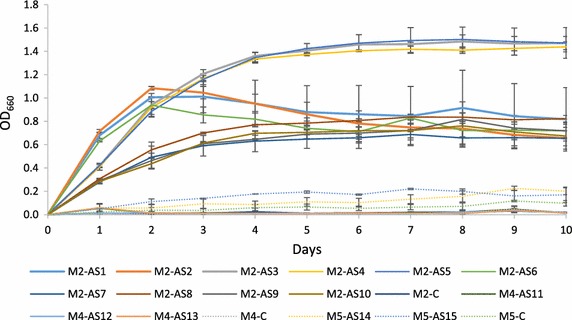
Bacterial growth assay (OD_660_). The data represents average of three independent experiments with SD values (±). Three different miocrobial density trends were observed in the medium M2: high density (AS3, AS4 and AS5), intermediate density, initial higher density (AS1, AS2 and AS6) and low density (AS7–AS10). The media M4 and M5 showed poor growth

The size of clearance zones in 150 petri dishes were measured after 20, 50 and 70 h of incubation (Fig. 2). Isolates AS1–AS10 from M2 outperformed the AS11–AS13 isolates from M4 and from M5 isolates (AS14 and AS15). M4 isolates performance were similar to M2 after 70 h; however, M4 isolates were inconsistent. Optical density (OD_660_) trends which represent microbial growth were measured as shown in Fig. 3. Three different OD_660_ were observed at the M2 isolates: high density (AS3, AS4 and AS5), intermediate density yet initially higher density (AS1, AS2 and AS6) and low density (AS7–AS10). M5 had low density, but M4 density was negligible. These density trends coincide with the observed biodegradation performance at the previous sections ensuring the suitability of the M2 medium. The pH values in all media (M2, M4 and M5) remained neutral during the course of experiment.

### Biodegradation**/**biotransformation under aerobic and anaerobic conditions

**Fig. 4:**
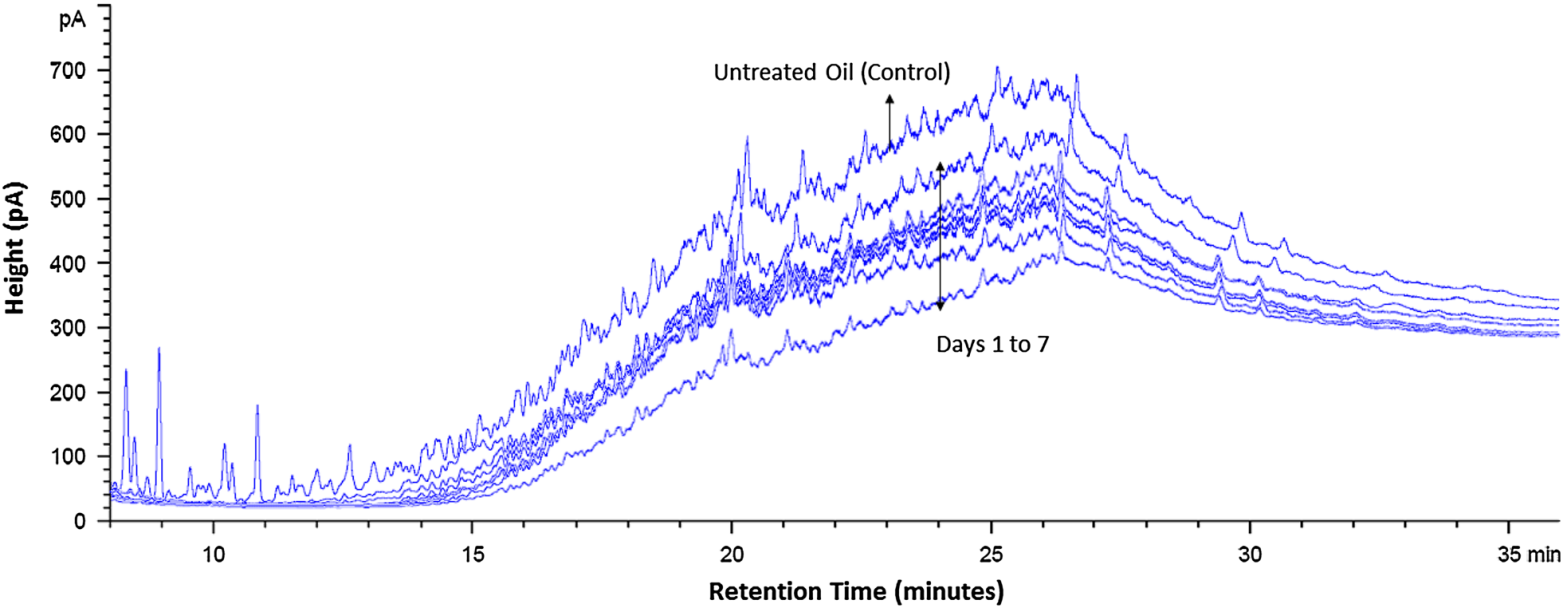
GC-FID chromatograms of heavy crude oil degradation by *B. licheniformis* AS5 isolate

**Fig. 5:**
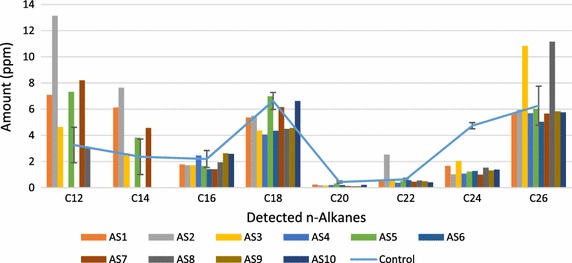
The degradation of heavy crude oil by isolates AS1–AS10 under aerobic conditions. More than 50 % of the C24 was degraded. C12 and C14 increase by several isolates indicated HCO biotransformation from heavier to lighter compounds

**Fig. 6:**
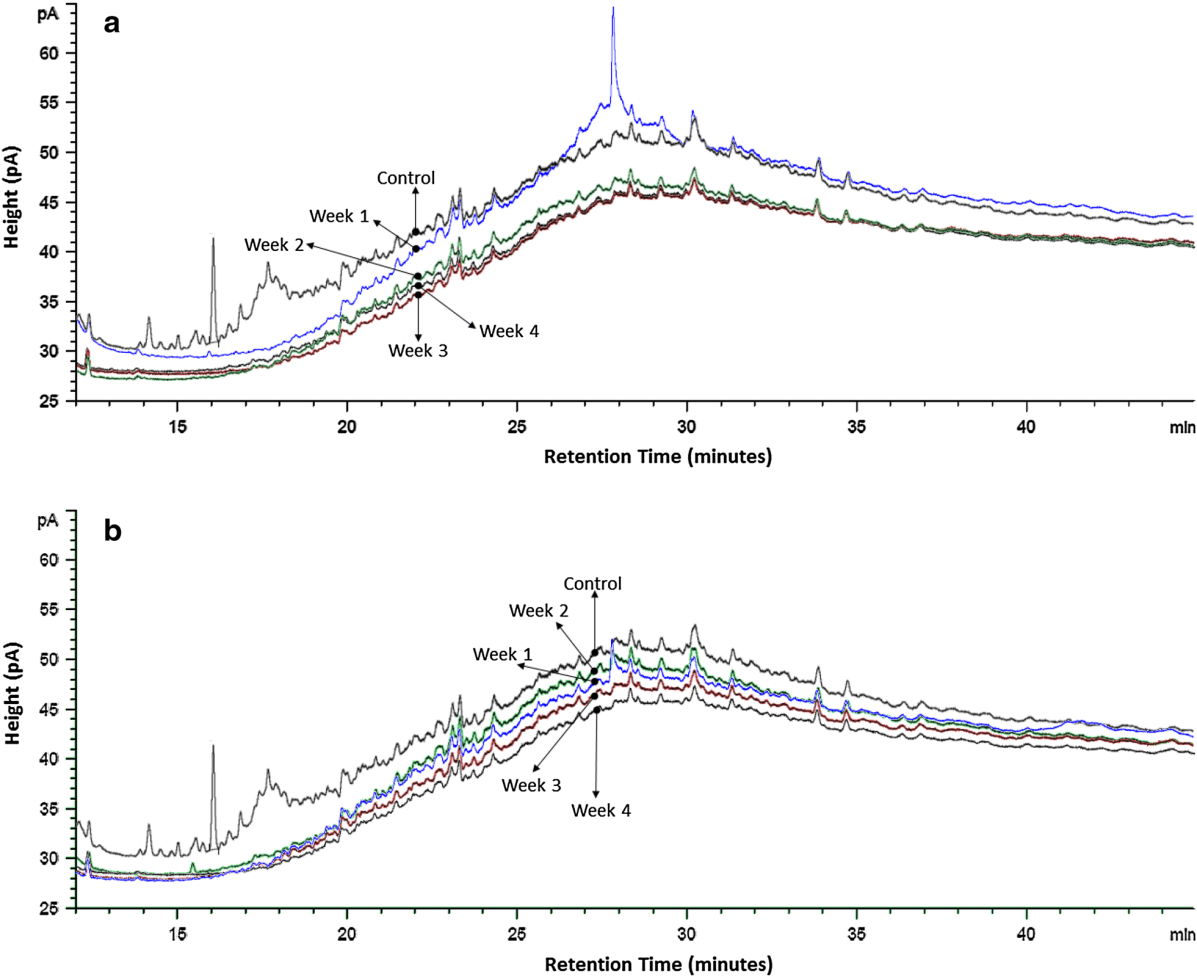
GC-FID chromatograms (**a**, **b**) of heavy crude oil degradation by isolates AS2 and AS5, under anaerobic conditions

Aerobic biodegradation and biotransformation of the HCO by isolate AS5 with the highest optical density was monitored daily for 7 days (Fig. 4). The chromatograms showed clear evidence of biodegradation. All isolates (AS1–AS15) were evaluated for their biodegradation according to carbon atom numbers after 30 days (Fig. 5). Mixed *n*-alkanes with carbon atom numbers were used as standards (ASTM^®^ D5442 C12-C60 Qualitative Retention Time Mix) for GC analyses. Biodegradation of mixed alkanes varied among the isolates. Biodegradation and biotransformation of HCO occurred by more than 50 % where C24 was dominant. C16 and C18 was degraded by most isolates. Also, C26 was degraded by all with the exception of AS3 and AS8. C20 and C22 (except at AS2) were not degraded. However, a significant increase of C12 and C14 by several isolates indicated HCO biotransformation from heavier to lighter compounds. *B. subtillis* AS2 and *B. licheniformis* AS5 were then tested for biodegradation and biotransformation ability under anaerobic conditions. The extracted hydrocarbon solution oil phase was analyzed by GC-FID and the gas phase was analyzed by GC-TCD. The weekly chromatograms analyses of GC-FID were conducted for 4 weeks (Fig. 6). Anaerobically, GC analyses of the mixed *n*-alkane standards were undetectable. GC-TCD analyses showed produced CO_2_ indicating degradation of hydrocarbons by the microbes.

### AS5 bacteria increased heavy crude oil recovery

**Fig. 7:**
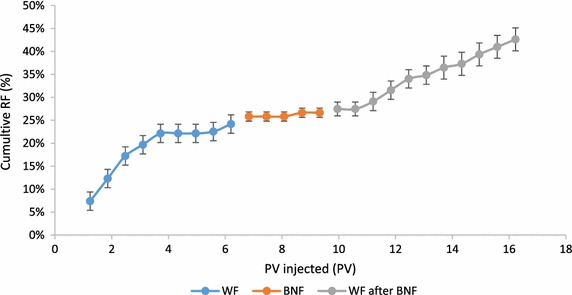
Cumulative recovery factor versus injected PV using *B. licheniformis* AS5 isolate. WF recovered = 24.2 % of OIIP, BNF = 2.5 % of OIIP recovered. WF after 5 days of incubation of isolate *B. licheniformis* AS5 resulted in an additional 16 % recovery

**Fig. 8:**
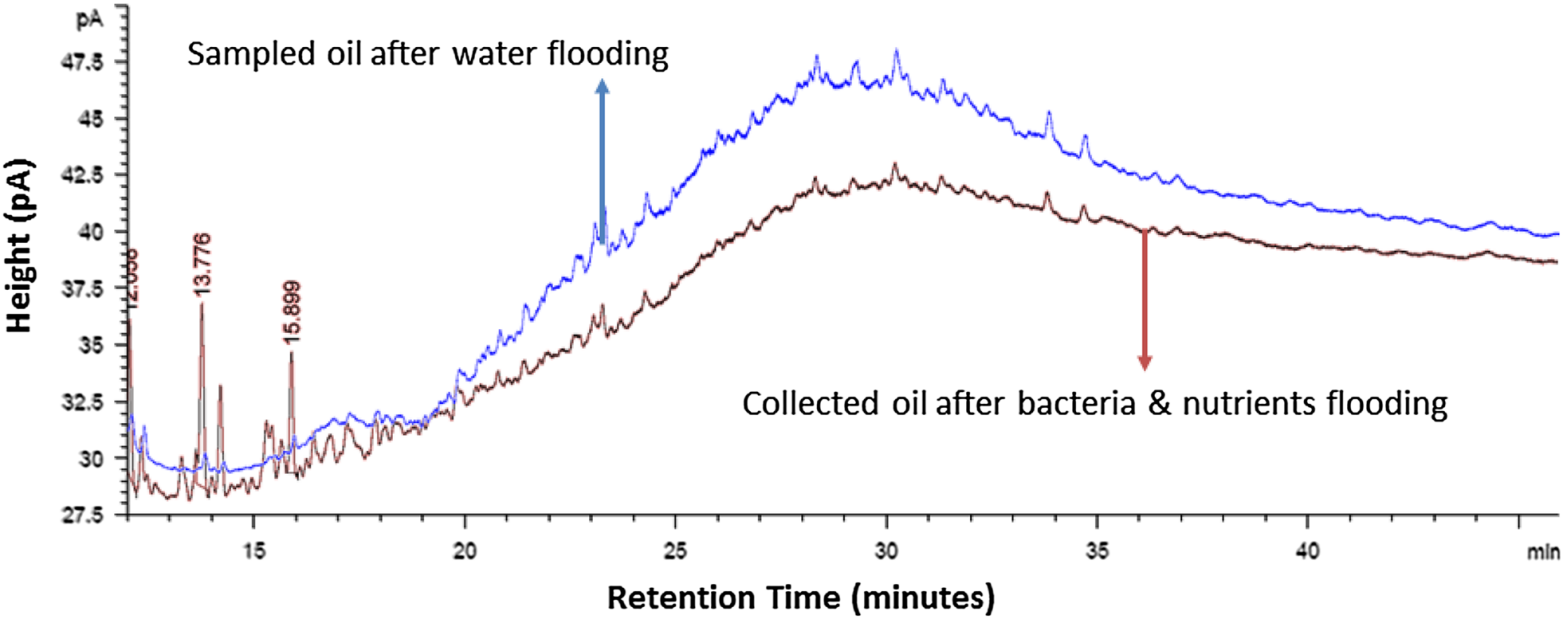
Chromatograms of heavy crude oil recovered from the initial water flood and from the water flood after microbial treatment

**Fig. 9:**
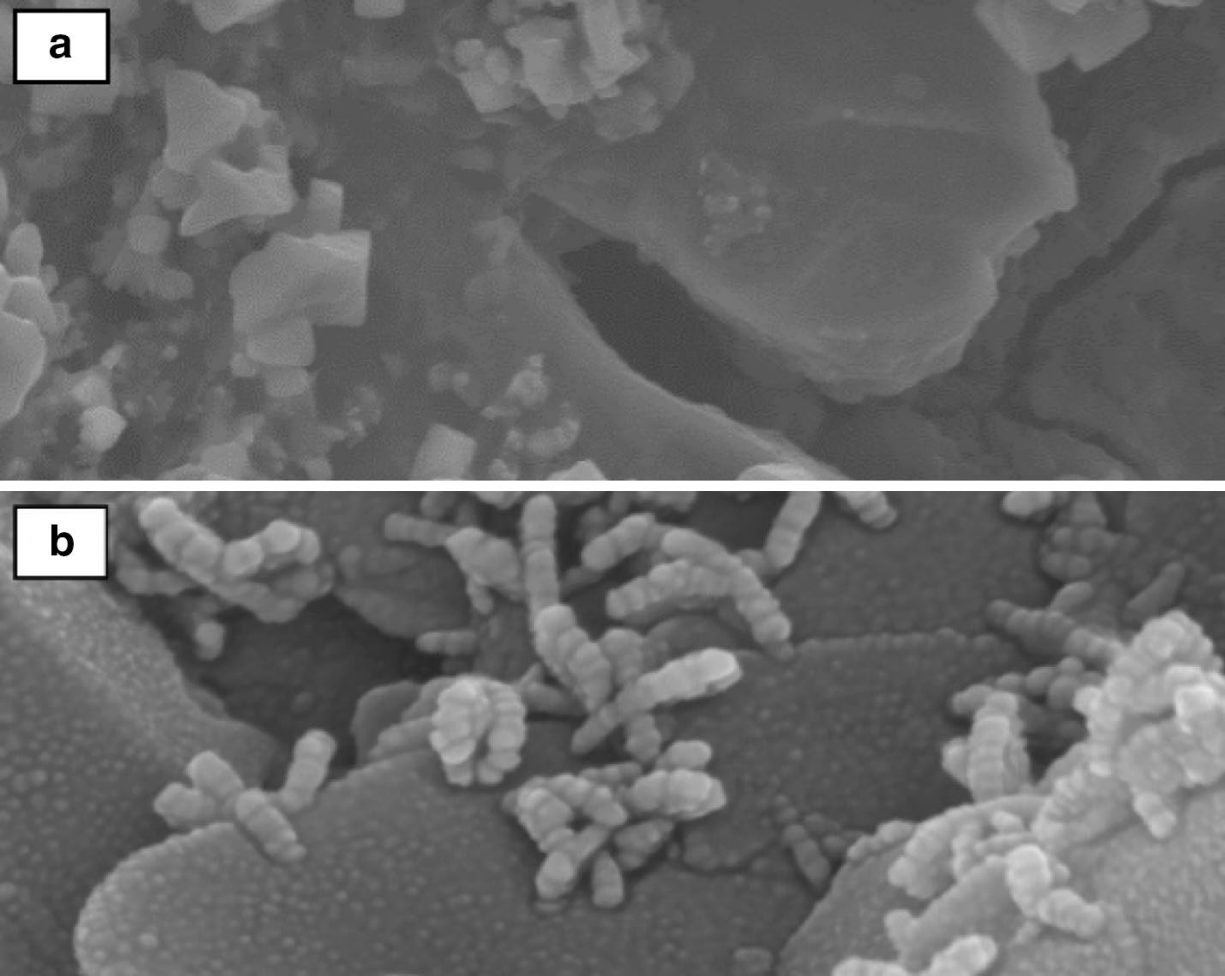
**a** SEM image of Berea core-plug at sterile conditions (control). **b** SEM image of the growth of *B. licheniformis* AS5 isolate in Berea core-plug after core-flooding experiment at 100,000 magnification, leading to additional heavy crude oil recovery

For the core flooding procedure using Berea sandstone core, 6.8 × 10^5^ CFU/mL of AS5 bacterial cells in early log phase were injected (5 %) after the water flood (WF). The core had 75.66 % initial oil saturation (S_oi_), 19.37 % porosity, 210.15 md Helium permeability (k), 7.4 cm length and 3.8 cm diameter. Percentage of recovery factor versus injected pore volume (1 PV = 16.12 cm^3^) was calculated (Fig. 7). WF recovered 24.2 % of initial oil in place (OIIP) where the trend started to rich a plateau indicating negligible recovery. The isolate AS5 with nutrients were injected in core, but only 2.5 % of OIIP recovered. After 5 days of incubation with AS5, the core was flooded with brine and additional 16 % of OIIP was recovered indicating the recovery effectiveness under anaerobic condition. The collected oil fractions from the water flood before and after the incubation with bacteria were analyzed by GC-FID (Fig. 8). The HCO biodegradation and biotransformation were evident by the increase in lighter compounds, as evident by GC. SEM analyses on the core after the flooding experiment also showed that isolate AS5 was able to grow under anaerobic conditions in core-plugs, leading to additional recovery (Fig. 9).

The results produced by this study are comparable with recent research findings in terms of increased crude oil recovery although our conditions were more challenging. A study reported 10.1 % additional recovery at core flooding experiments although non-heavy crude oil and a consortium of bacteria were used where volatile fatty acids, organic acids, surfactants, exopolysaccarides and CO_2_ at 91 °C in optimized nutrient medium were bio-produced [17]. Another study found a single strain applied on a non-heavy oil recovered less than 5 % at core flood experiments [18]. The researchers explained that bioproducts by the AR80 strain were not observed thus the additional oil recovery was solely caused by a decrease in oil viscosity thru degrading long-chain *n*-alkanes to shorter ones. S17 and S28 strains in a different study conducted on a crude oil of 73 cP viscosity at 59 °C were able to decrease viscosity by 37 and 35 %, respectively. The heavy components of crude oil were degraded to light components and some biosurfactants were produced which were attributed to the reduction of viscosity. The bacteria achieved 12.26 % additional recovery [9]. At our study, a single bacterial strain and a much heavier crude oil was used (968 cP and at 40 °C) and AS5 did not produce any biosurfactacts. Thus, there is significant potential to be explored with optimized M2 medium conditions and applying a consortium of bacteria.

## Conclusion

Several spore forming bacteria capable of growing in M2 minimal medium containing only heavy crude oil as a carbon source were effectively able to degrade and transform HCO resulting in improved oil characteristics. During microbial growth, biotransformation of HCO to lighter hydrocarbons increased significantly as evident by well assays on oil containing agar plates and further confirmed by GC. Locally isolated *B. licheniformis* AS5 showed promising results as it showed additional 16 % extra heavy oil recovery after 5 days incubation at 40 °C. There is significant potential to be explored with optimized M2 medium conditions and nutrient sources; besides, applying a consortium of bacteria could enhance biodegradation and recovery even further. The results establish that inexpensive and environmentally friendly biodegradation process is an option for utilizing heavy crude oil resources. Locally isolated bacteria do not pose a risk to the local ecosystem and provide sustainability to biologically-based projects in the country.

## Methods

### Samples and media

Four oil contaminated soil samples, labeled as S1–S4 (weight 1.8 kg S1, 2.0 kg S2, 1.8 kg S3 and 1.4 kg S4) were collected randomly from a HCO field sludge pits. After removing 15 cm of the pit top layers, the collected samples were kept in plastic bags and stored at room temperature. The HCO was provided by the Petroleum Development of Oman (PDO). Oil viscosity, density, API gravity and asphaltene content were determined.

Five different media, including Bushnell Haas, were used for microbial growth (compositions are illustrated in Table 1) [11, 14, 19–21]. The trace elements and vitamin solution of M2 were replaced with the following trace elements/concentrations (g/L): ZnSO_4_**·**7H_2_O, 2.32; MnSO_4_**·**4H_2_O, 1.78; H_3_BO_3_, 0.56; CuSO_4_**·**5H_2_O, 1.00; Na_2_MoO_4_**·**2H_2_O, 0.39; CoCl_2_**·**6H_2_O, 0.42; EDTA, 1.00; NiCl_2_**·**6H_2_O, 0.004 and KI, 0.66. HCO was the sole carbon source in all experiments. Yeast extract in M3 was initially eliminated and was added later on to check the yeast’s effect on biodegradation and biotransformation processes. Uninoculated sterile agar and broth media were used as controls to check and confirm that HCO is being degraded by bacteria only.

### Isolation of bacteria

**Fig. 10:**
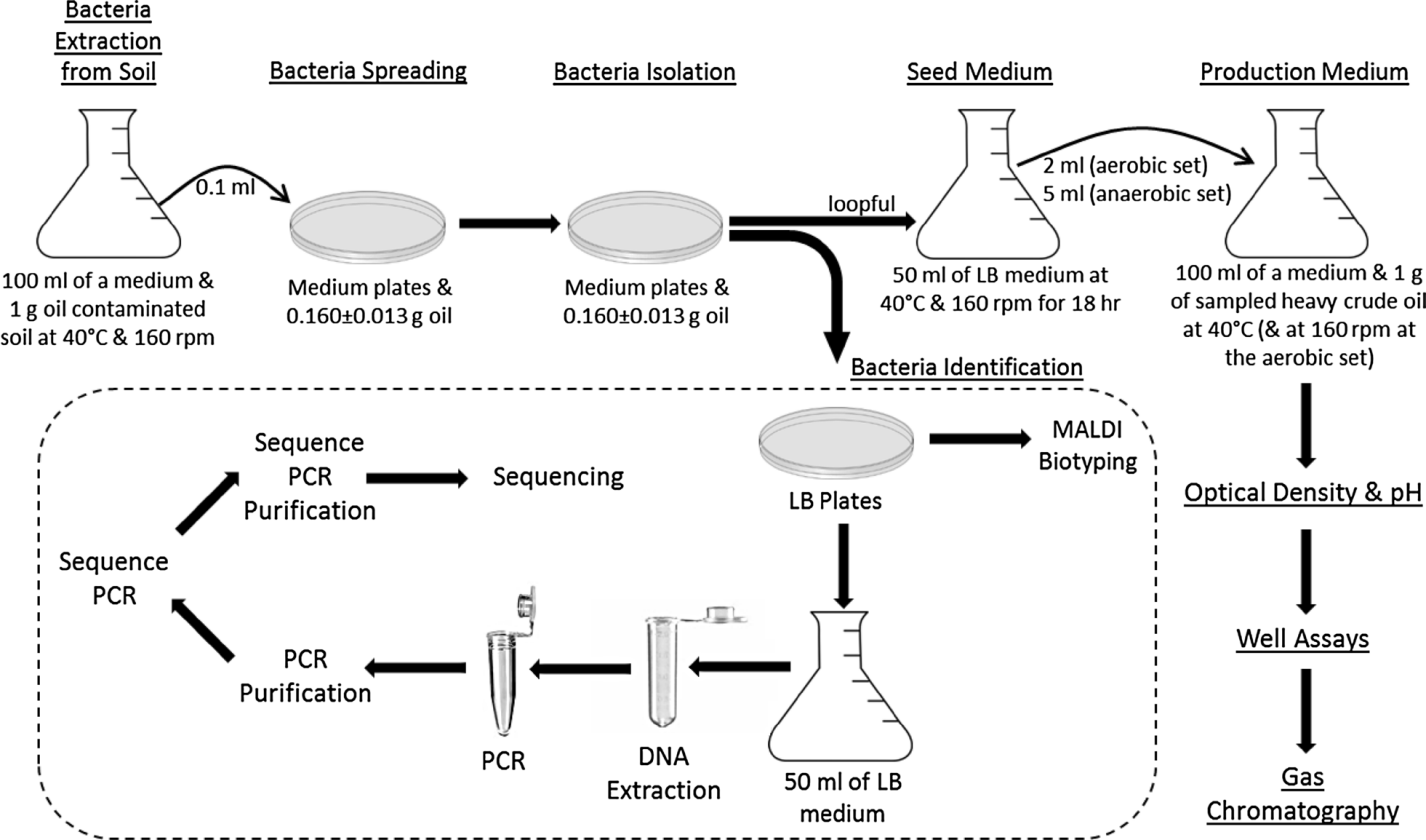
Schematic summary of the procedures followed for isolation, identification and screening of heavy crude oil degrading bacterial isolates

One gram of each oil-contaminated soil sample (1 and 2) were added separately to 250 mL flasks with 100 mL M1 to M5 media without heating to test for spore forming and non-spore forming bacteria capability to degrade HCO. The flasks were incubated in a shaker set at 160 rpm at 40 °C for 1 week. Five drops (0.160 ± 0.013 g) of sterile autoclaved HCO and 0.1 mL aliquots were added to each flask and spread on petri dishes. A streaking plate method for isolation single colonies was used. One gram of oil-sludge samples (3 and 4) were placed in 250 mL flasks containing 100 mL distilled water and separately heated at 80 °C in water bath for 30 min in order to eliminate non-spore forming bacteria. The steps taken at samples 1 and 2 were then repeated for samples 3 and 4. Control flasks and petri dishes were prepared for all media. The procedures are summarized schematically at Fig. 10.

### Identification of bacterial isolates

Each bacterial colony was grown in 5 mL Luria–Bertani (LB) medium and incubated at 40 °C for 18–24 h. Swabs were then used to transfer the strains to the MALDI biotyper (Bruker MALDI Biotyper CA System) for identification. Strain identification was based on four categories using the provided MALDI biotyper database: highly probable species, secure genus, probable species and unreliable identifications.

For the molecular sequencing, DNA was extracted by using the UltraClean Soil DNA Isolation Kit (MOBIO Laboratories) following MO BIO Laboratories’ procedure. Analysis of the 16S rRNA gene was performed for the taxonomic characterization of the isolated bacteria. The 16S rRNA was amplified using the domain-specific universal forward primer 27F (5′-AGAGTTTGATCCTGGCTCAG-3′) and the universal reverse primer 1492R (5′-AAGGAGGTGATCCAGCCGCA-3′) at 10 pmol/μL concentration. The amplification reaction (Polymerase Chain Reaction; PCR), was performed on a total volume of 25 μL containing: 12.5 μL master mix (Taq polymerase and dNTP mix), 9.5 μL double distilled (D.D.) H_2_O, 1 μL extracted DNA and 1 μL of each primer. PCR amplification was performed with initial denaturation step at 94 °C for 3 min followed by 35 cycles of 1-min denaturation step at 94 °C, 2 min annealing step at 53 °C and 2 min elongation step at 72 °C, and a final extension step at 72 °C for 7 min using a 2720 thermal cycler (Applied Biosystem). The PCR products were detected in 1 % agarose gel electrophoresis. The PCR products were purified using QIAquick PCR purification kit (QIAGen). A second PCR reaction (sequence reaction) was carried out using 0.7 μL of the first PCR product as a template, 2 μL of BigDye^®^ terminator, 2 μL of 5× sequence buffer, 1 μL of 3 pmol/μL a primer and 4.3 μL of D.D. H_2_O (making a total volume of 10 μL). 27F, 515F and 1492R primers were used, each at a separate PCR tube along with the other constituents. The sequence PCR amplification was performed as per the following program: a single cycle at 96 °C for 20 s, 60 cycles of 96 °C for 10 s, 50 °C for 5 s and 60 °C for 4 min, and cooling at 4 °C. The sequence reaction was then purified using the DyeEx 2.0 spin kit (QIAGen). The purified sequence reaction products were sequenced using 3130 XL Genetic Analyzer (Applied Biosystem, Hitachi).

Sequence alignment of the three sequences per bacterial strain was performed using the maximum likelihood method of Hasegawa–Kishino–Yano model [13].The aligned sequences of the bacterial strains were submitted to GenBank NCBI. The tree with the highest log likelihood was −2378.5282. neighbor-join and BioNJ algorithms to matrix of pairwise distances were found automatically for the initial heuristic search. The maximum composite likelihood (MCL) was used by the topology with superior log likelihood value. The rate variation model for some sites evolutionarily constant was [+I], 18.8 % sites. The phylogenetic tree was scaled by measuring the number of substitutions per site with branch lengths. The analysis involved 30 nucleotide sequences. Positions below 95 % site coverage were not used. Alignment gaps with less than 5 %, missing data and ambiguous bases were aligned at any position. There were a total of 1314 positions in the final dataset. MEGA6 was used for evolutionary analyses [22].

### Growth and biodegradation assay

For the well assays test, daily aliquots of the 15 isolated bacteria were taken for 10 days from their respective broth media. Four wells per agar Petri plate of 0.5 cm diameter were aligned diagonally with two test wells and two control wells. Microbial aliquots were loaded into the wells. After 20, 50 and 70 h of incubation, clearance zones were measured around the wells. To assess growth of the isolated bacteria, 2 mL samples were taken daily from each broth to measure optical density at 660 nm (OD_660_). Any changes in samples’ pH were assessed daily to detect any changes during biodegradation.

### Gas chromatography (GC)

LB broth was used as seed medium. Bacteria in LB seed medium were inoculated in flasks containing 100 mL of M2 medium and 1 g of the HCO. M2 flasks were incubated at 40 °C in shaker set at 160 rpm and were analyzed daily for a week. Heptane was added to the media flasks (1:10 oil to heptane). HCO and heptane solution was separated from the media by a separatory funnel.

Biodegraded and bio-transformed HCO was analyzed by gas chromatography (GC) with flame ionization detector (FID) (7890A GC System, Agilent Technologies, USA. HP-1 column (Agilent Technologies, USA).Nitrogen gas carrier was used at a flow rate of 4 mL/min. The oven temperature was increased from 90 to 300 °C at a rate of 15 °C/min and kept at the final temperature for 44 min.

Evolved gases under anaerobic conditions were analyzed by GC with thermal conductivity detector (TCD) (6890N GC System, Agilent Technologies, USA). HP-PLOT/Q column (Agilent Technologies, USA) and was used for the GC-TCD analysis, and nitrogen carrier gas was used at a flow rate of 4 mL/min. The oven temperature was increased from 50 to 80 °C at a rate of 20 °C/min and kept at the final temperature for 8.5 min.

### Enhanced oil recovery experiments using core flood

The viscosity and density of the sampled HCO were measured at 968 cP and 0.961 g/cc, at 40 °C, respectively. The measured asphaltene content (%), as per the IP143 standard, and the API gravity of the crude were 5.5 % and 13.3° API, respectively. HCO without microbes (controls) incubated at 40 °C in a shaker set at 160 rpm, after 20 days did not show any degradation.

Berea sandstone cores were cleaned by using the soxhlet extraction method with methanol as the solvent and dried at 65 °C for 24 h before usage. The cores were saturated with filtered formation brine using vacuum desiccators and pore volume (PV) was determined using the dry and wet weights of the cores. The cores were flooded with oil at 0.4 cm^3^/min until no more water was produced. The OIIP which was indicated by the volume of water displaced was determined. The core was subjected to WF at 0.4 cm^3^/min. The residual oil was calculated by measuring the amount of oil produced from the waterflood. 1.5 PV of 5 % bacterial solution in LB seed medium was inoculated in M2 production medium and was then injected into core flood. The tertiary recovery of extra oil was calculated. Core floods were conducted at 40 °C. CFU/mL of bacteria per 1 mL was estimated by serial dilution method after incubation for 18 h in LB broth at 40 °C placed in a shaker set at 160 rpm. The inoculants (0.1 mL) were spread onto LB agar and incubated at 40 °C for 1 day. Recovered HCO by water flooding before and after incubation with bacteria was analyzed by GC-FID. Scanning electron microscopy (SEM) was conducted to visualize the bacteria at the core after the flooding tests.
